# Reply to David et al, “Studying *Pseudomonas aeruginosa* pathogenesis: the need for more than just the PAO1 strain”

**DOI:** 10.1128/spectrum.01177-25

**Published:** 2025-08-11

**Authors:** Jing Ge, Min Cao, Yuyao Zhang, Tianqi Wu, Jiayi Liu, Jiang Pu, Hongye He, Zhibin Guo, Shaoqing Ju, Juan Yu

**Affiliations:** 1 Department of Laboratory Medicine, Affiliated Hospital of Nantong University 74567, Nantong, China; 2 Medical School of Nantong University 117814, Nantong, China; 3 Krieger School of Arts and Science, Johns Hopkins University 1466, Baltimore, USA; 4 Institute of Public Health, Nantong University, Nantong, China; University of Florida, Gainesville, Florida, USA

## REPLY

Thank you for the opportunity to respond to the thoughtful comment by Dr. David and colleagues. We sincerely appreciate their engagement with our recent study, *“*Inhibiting NLRP3 enhances cellular autophagy induced by outer membrane vesicles from *Pseudomonas aeruginosa,*” and their valuable insights, which underscore the importance of rigorous scientific discourse.

In the initial stage of research, researchers need to first establish causality in a simplified system and then consider the hierarchy of biological complexity. Indeed, many breakthrough discoveries in bacterial pathogenesis have followed this approach (1). Our study follows this scientific tradition and uses PAO1 as a “microbial model system” to establish a conceptual framework for the study of the interaction between outer membrane vesicles (OMVs), autophagy, and inflammasome activation in host cells. PAO1 was selected based on its well-established role in *Pseudomonas aeruginosa* research, owing to its comprehensively annotated genome, genetic tractability, and extensive characterization in prior literature (2–4). As noted by Dr. David and colleagues, PAO1 encodes a truncated version of the CprA protein, which may result in OMVs with different properties than those produced by strains encoding full-length CprA.

We fully acknowledge the phenotypic and genomic diversity across *P. aeruginosa* strains and agree that findings derived from PAO1 should not be overgeneralized. However, our intention was not to suggest a universal OMV phenotype but rather to explore a mechanistic connection on how PAO1-derived OMVs modulate autophagy and how this process is influenced by NLRP3 inflammasome signaling. The enhancement of autophagic flux via NLRP3 inhibition, as observed in our study, represents an important biologically meaningful finding within this defined context. We agree that future investigations incorporating a broader range of *P. aeruginosa* strains, such as those with functional CprA, will be essential. Nevertheless, we believe our current study provides useful insights and establishes an important foundation for future comparative analyses across diverse isolates. We aimed to keep the title concise without sacrificing clarity, in line with most scientific communication practices, as we emphasized the specific use of strain PAO1 throughout the text. However, we fully agree that the applicability of these findings will need to be validated in different clinical isolates. This exemplifies the full scientific path from model development to clinical validation. Thank you very much for the helpful reminder and suggestions to provide further clarifications where appropriate.

The observation about CprA’s role in autophagy flux is indeed insightful, and we appreciate Dr. David highlighting this aspect. Their work and discussion add depth to the scientific dialogue surrounding *Pseudomonas aeruginosa* pathogenesis.

Once again, we deeply appreciate Dr. David’s attention to our work. We hope this exchange benefits the broader research community’s efforts in understanding OMV-mediated host-pathogen interactions.
